# The Relationship Between Physical Exercise and Mobile Phone Addiction Tendency of University Students in China: A Moderated Mediation Model

**DOI:** 10.3389/fpsyg.2022.730886

**Published:** 2022-02-14

**Authors:** Ke-lei Guo, Qi-shuai Ma, Shu-jun Yao, Chao Liu, Zhen Hui, Juan Jiang, Xi Lin

**Affiliations:** ^1^School of Physical Education and Health, Zhaoqing University, Zhaoqing, China; ^2^School of Physical Education, Huaibei Normal University, Huaibei, China; ^3^School of Marxism, Zhaoqing University, Zhaoqing, China; ^4^School of Wushu and Dancing, Shenyang Sport University, Shenyang, China; ^5^School of Physical Education and Health, Longyan University, Longyan, China

**Keywords:** physical exercise, mobile phone addiction tendency, self-control, physical exercise atmosphere, university students, China

## Abstract

This study aims to explore the relationship between physical exercise (PE), self-control (SC), physical exercise atmosphere (PEA), and mobile phone addiction tendency (MPAT) among Chinese university students. Through the quota sampling, 1,433 students complied with the requirements were surveyed from 10 universities in China. PE, SC, PEA, and MPAT were assessed using standard scales. For data analysis, common method deviation test, mean number, standard deviation, correlation analysis and structural equation model analysis were carried out in turn. The results showed PE and MPAT were negatively related (*r* = –0.158, *p* < 0.05); PE significantly positively predicted SC (β = 0.082, *t* = 3.748, *p* < 0.01), and SC significantly negatively predicted MPAT (β = –0.743, *t* = –19.929, *p* < 0.01). Bootstrap method was used to test the mediating effect of SC. The results showed that 95% confidence interval did not include 0. After adding the mediating variable of SC, PE did not significantly negatively predict the tendency of MPAT (β = –0.027, *t* = –1.257, *p* > 0.05). The interaction item PEA and SC could significantly positively predict the tendency of MPAT (β = 0.165, *t* = 2.545, *p* < 0.05). In the high PEA group, SC had a significant negative predictive effect on the tendency of MPAT (β = –0.665, *t* = –14.408, *p* < 0.01); However, in the low PEA group, the negative predictive effect was stronger (β = –0.834, *t* = –15.015, *p* < 0.01). The present study shows that PE significantly negatively predicted the tendency of MPAT, and SC played a complete mediating role in the relationship between PE and MPAT; The second half of the indirect effect of PE and MPAT was regulated by the PEA. The PEA will enhance the influence of SC on MPAT, but the high PEA will increase the level of MPAT of individuals at a very high level of SC.

## Introduction

In recent years, the rapid development of the Internet and other types of information and communication technology has brought exponential growth to mobile phone users (Toda et al., 2006; Xavier et al., 2018). Many functions of applications related to the Internet of mobile phones, especially smart phones have become the principal use of mobile phones, replacing traditional calling and information transmission methods (Yang et al., 2019). According to a report from the China Internet Network Information Center, the number of mobile internet users in China had increased to 932 million by June 2020, accounting for 99.2% of the total internet users (CNNIC, 2020). Among mobile phone netizens, it is apparent that young adults (aged 18–25 years) have become the largest group of users, making up an incredible ratio of more than one third of users (Yang et al., 2019). The majority among this group might be surmised to be university students.

As a very practical and multifunctional digital device, mobile phones are widely employed by university students (Yali et al., 2020). They apply mobile phones to various kinds of aspects in daily life, including shopping online, playing games, watching videos, browsing journals, communicating with others and instant buying or paying (Servick, 2015). However, it should be considered that excessive mobile phone usage might do more harm than good and is even likely to cause some serious passive consequences, such as mobile phone addiction tendency (MPAT). Mobile phone addiction tendency refers to the obsessive state in which individuals’ psychological, physiological and social functions are obviously damaged due to the loss of control of mobile phone use behavior (Yen et al., 2009). Research shows that about a quarter of Chinese university students are exposed to the problem of mobile phone addiction (Huan et al., 2014; Huihui et al., 2015). As for university students, mobile phone overuse not only leads to a poor academic performance, but can also contribute to some mental disorders such as depression, social anxiety, stress, and insomnia as well as negative emotions in general (Hong et al., 2012; Haruka et al., 2017; Aleksandar et al., 2018).

### Physical Exercise and Mobile Phone Addiction Tendency

Apart from the detrimental effects on learning and mental health, excessive mobile phone use can disrupt university students’ physical activity to a large extent (Andrew et al., 2013), as supported by several previous Findings (Kim et al., 2015; Barkley and Lepp, 2016; Penglee et al., 2019). Kim et al. (2015) found that smart phone addiction among university students may negatively influence physical health by reducing the amount of time spent in physical activity such as walking. Penglee et al. (2019) revealed that excessive utilization of smart phone among college students may be a barrier to physical activity and proposed strategies to promote physical activity in higher education environments. Similarly, Barkley and Lepp (2016) pointed out that the act that college students use mobile phones can be viewed as a sedentary leisure behavior resulting in poor physical activity. Therefore, based on the above research, we put forward the hypothesis 1: PE and MPAT may be negatively correlated with each other.

### The Mediating Role of Self Control

Some previous studies have proven the positive relationship between PE and SC, including different intensities (Sibley et al., 2006; Kamijo et al., 2007), different periods (Tomporowski et al., 2008; Davis et al., 2011), and different types of physical activities and exercise programs (Caterina et al., 2009; Davis et al., 2011). Sibley et al. (2006) found that moderate intensity aerobic exercise could improve the inhibitory function of university students, which was an indispensable component of SC, but Kamijo et al. (2007) pointed out that high intensity physical exercise may not strengthen individuals’ SC. Both Tomporowski et al. (2008) and Davis et al. (2011) indicated that both acute and chronic physical exercise can be beneficial to the enhancement of SC. Davis et al. (2011) suggested that group activities such as football or basketball can also effectively improve SC. In addition, several personality traits have been found to be associated with the extent of mobile phone usage, such as self-esteem, self-regulation, and self-control (SC) (Khang et al., 2013; Andrew et al., 2014). Compared to self-esteem and self-regulation, SC has been verified to be a more important psychological predictor for problematic mobile phone use, showing a negative association between the two (Mi and Sohn, 2014). High self-control has been proved to be a highly indispensable protection factor against Internet addiction in adolescents. Nevertheless, facing stimuli and temptation from the visual world, youth with low self-control are extremely prone to the harm of Internet addiction (Li et al., 2013; Jianhui, 2015). Considering the likely correlation between SC and MPAT, the improvement of SC might imply a decrease of MPAT. Yang et al. (2019) believed that SC played a significant mediating role between PE and mobile phone dependence. Based on the above researches, we put forward the hypothesis 2: SC would play a mediating role between PE and MPAT.

### The Moderating Role of Physical Exercise Atmosphere

According to the theory of Individual-environment Interaction, individual behavior problems are the result of interaction between individual factors and environment (Lerner et al., 2006). Therefore, the individual’s mobile phone addiction tendency may also be regulated by the environmental system. Physical environment (including family physical environment, school physical environment, and community physical environment) has an important impact on individual development, especially the physical exercise environment felt by students. Physical exercise atmosphere (PEA) refers to the situation of people around an individual participating in physical exercise and the sports-related media information that an individual is exposed to, usually including natural atmosphere and interpersonal atmosphere (Weina et al., 2011; Yunfeng and Baolin, 2018). Compared with other physical environments, the impact of physical exercise atmosphere on students is more direct (Jialin et al., 2017).

According to the Self-determination Theory, human beings have three basic psychological needs: relationship needs, autonomy needs and ability needs. Whether the external environment can meet the basic psychological needs of individuals plays an important role in their mental health and personal growth (Deci and Ryan, 1985, 2000; Ryan and Deci, 2017, 2020). Individuals with low SC level can not meet their belonging needs of school and family, and lack self-confidence and self-identity (Yingmin et al., 2021). A good PEA can stimulate students’ desire to exercise and improve their enthusiasm for participation (Ronghua et al., 2017), to form physical exercise habits (Xiugang et al., 2010), and to help university students establish group identity and behavioral identity in PE. It can also help university students build self-confidence and self-identity (Yu and Yong, 2006). If these meet the basic psychological needs of individuals with low SC level, it will be beneficial to their benign development and the reduction of the MPAT. Previous studies have shown that individuals’ perception of good friendship and PEA is conducive to reducing MPAT and other internalization problems (Pengcheng et al., 2017; Yongxin et al., 2018), enhancing emotional and social adjustment (Gazelle, 2006). Based on the above studies, we put forward the hypothesis 3: Physical exercise atmosphere would moderate the relationship between SC and MPAT.

### The Present Study

**FIGURE 1:**
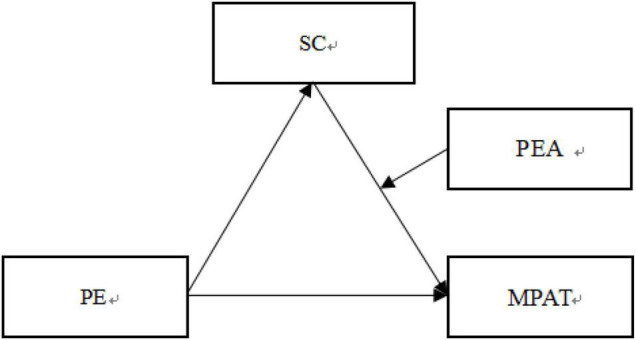
The proposed moderated mediation model.

Based on the literature review above, the study constructed a moderated mediation model to examine the mediating role of SC in the relationship between PE and MPAT among Chinese university students. Furthermore, it was tested whether the indirect path between SC and MPAT would be moderated by PEA. The proposed theoretical model was illustrated in Figure 1.

## Materials and Methods

### Participants and Procedure

Participants were recruited from 10 universities (including Science Universities, Liberal Arts Universities, and Comprehensive Universities) in central China. Stratified cluster sampling method was adopted to randomly select 1 class in each grade of each school to issue questionnaires. A total of 1,550 students were approached, and 1,433 valid questionnaires were recovered (117 participants were excluded for the missing data on the main variables). Among the participants, there were 704 boys and 729 girls, 374 freshmen, 363 sophomores, 335 juniors, and 361 seniors, with an average age of 19.67 ± 1.62.

The main tests were all professionally trained students majoring in Sports Psychology, with the consent of their college leaders, teachers and the subjects themselves. The collective test was adopted and the principles of Voluntary Filling, Data Confidentiality and Anonymous Filling were applied. Variables such as gender and grade of the participants were controlled as well. Furthermore, the research design was approved in an ethical review process conducted by the Human Research Ethics Committee of Zhaoqing University. The data collection was based on a project aiming at the investigation of college students’ mental health during 21 December 2020 and 18 February 2021. This study took the respondents approximately 5–10 min to complete all the questionnaires, during which all invited participants were voluntary and guaranteed confidentiality.

### Measures

#### Physical Exercise Atmosphere

Physical exercise atmosphere was evaluated by the Physical Exercise Atmosphere Scale (PEAS) (Shanping and Shuzhuo, 2007). The PEAS is a five-point Likert scale with eight items and two dimensions: cultural atmosphere and activity atmosphere. Each item is scored from 1 (completely disagree) to 5 (completely agree), and the total score can be from 8 to 40. A higher score indicates a deeper degree of PEA. Both exploratory and confirmatory factor analysis have supported the construct validity of the two dimensions. The internal consistency coefficient of PEAS is 0.70, and the test–retest reliability is 0.84 (Shanping and Shuzhuo, 2007). Additionally, a previous study demonstrated that this scale was applicable among college students (Baolin and Lijuan, 2018). In this study, Cronbach’s alpha was 0.83.

#### Mobile Phone Addiction Tendency

Mobile phone addiction tendency was assessed by the mobile phone addiction tendency scale (MPATS) (Xiong et al., 2012). The MPATS is a five-point Likert scale with 16 items and four dimensions: withdrawal symptoms, salient behavior, social comfort, and mood change. Each item is scored from 1 (completely inconsistent) to 5 (completely consistent), and the total score can vary from 16 to 80. A higher score indicates a deeper degree of MPAT. Both exploratory and confirmatory factor analysis have supported the construct validity of the four dimensions. The internal consistency coefficient of MPATS is 0.83, and the test–retest reliability is 0.91 (Xiong et al., 2012). Moreover, a previous study demonstrated that this scale was applied well in the sample of college students (Juan et al., 2021). For this study, Cronbach’s alpha for this scale was 0.89.

#### Self-Control

The SC was measured by the self-control scale (SCS) (Tan and Guo, 2008), which was modified based on Tangney’s Self-Control Scale (Tangney et al., 2004). The SCS is a five-point Likert scale and comprises 19 items. It has five dimensions: keeping healthy habits, controlling impulses, resisting temptation, concentrating on work and controlling entertainment. Each item is valued from 1 (completely disagree) to 5 (completely agree). The total score can vary from 19 to 95, and a higher score shows a higher level of individual self-control. The internal consistency coefficient of SCS is 0.86, and test–retest reliability is 0.85. Additionally, a previous study proved that this scale was reasonable in the sample of college students (Jiang and Zhao, 2016). In this study, Cronbach’s alpha was 0.90.

#### Physical Exercise

Physical exercise was evaluated by the Physical Activity Rating Scale-3 (PARS-3) (Deqing and Shaojun, 1994). The PARS-3 is a three-item self-reported scale, containing exercise time, exercise intensity and exercise frequency. Each item is rated from 1 to 5, and the total score of physical activity is computed by the following equation: intensity × (time–1) × frequency, with a range of 0 to 100. A total score that is equal to or less than 19 is defined as light, one that is 20 to 42 is defined as moderate, and one that is equal to or more than 43 is defined as vigorous physical activity. According to previous experience (Xiangwei et al., 2018), the current study divided the light physical activity into two components: no and light physical activity. No physical activity is equal to or less than 4, and the light physical activity ranges from 5 to 19. Thus, PE in this study was divided into four levels, from 1 (no physical activity) to 4 (vigorous physical activity). The PARS-3 has a fair internal consistency coefficient (α = 0.639) and test–retest reliability (*r* = 0.82). Moreover, a previous study proved that this scale was fairly applicable in the sample of college students (Yang et al., 2019). For this study, Cronbach’s alpha for this scale was 0.71.

### Statistical Analyses

First, all statistical analyses were performed with IBM SPSS statistical software (version 21.0) and Mplus 8.0 for Windows (SPSS, Chicago, IL, United States). Second, IBM SPSS statistical software version 21.0 was used to test the data for Harman Common Method deviation, and the relationship between PE, MPAT, SC, and PEA was calculated by Pearson’s correlation analysis. Continuous variables with normal distribution were presented as the mean ± standard deviation (SD). Third, in order to verify the mediating role of SC in the relationship between PE and MPAT, and whether this mediating process is regulated by PEA. According to the steps proposed by Wu Yan to test the moderated mediation model (Yan and Zhonglin, 2011): (1) The direct effect was tested between PE and MPAT; (2) Whether the direct effect was regulated by PEA was tested; and (3) MPLUS8.0 was used to conduct structural equation model analysis to test whether the mediating effect of SC during PE on MPAT was moderated by PEA. According to past experience, goodness of fit index χ^2^/DF less than 3 is acceptable, RMSEA less than 0.08, NNFI and CFI greater than 0.9, and SRMR less than 0.05 are considered acceptable, and in this study, the significance level was set at *p* < 0.05.

## Results

### Common Method Deviation Test

Firstly, Harman Single-factor Test was used to test the common method deviation (Podsakoff et al., 2003; Hao and Lirong, 2004), and it was found that there were 10 factors with eigenvalues greater than 1. The first factor could explain 19.23% of the variation, which was less than the standard threshold value of 40%. This result indicated that the common method deviation in this study was acceptable. The single common method factor control method was used to test the common method deviation, which was relatively accurate (Xiong et al., 2012). The results showed that the model containing the common method factors could not fit the data. Therefore, in this study, the results of both methods indicate that there is no obvious common method bias problem.

### Descriptive Statistics and Correlation Coefficients for Each Variable

As shown in Table 1, the correlation coefficients of PE, PEA, MPAT, and SC were all statistically significant. The PE was negatively correlated with MPAT (*r* = –0.158, *p* < 0.01), but positively associated with SC (*r* = 0.212, *p* < 0.01), as well as PEA (*r* = 0.338, *p* < 0.01). The SC was negatively correlated with MPAT (*r* = –0.607, *p* < 0.01), but positively associated with PEA (*r* = 0.248, *p* < 0.01). The PEA was negatively related to MPAT (*r* = –0.138, *p* < 0.01).

**TABLE 1:** Descriptive statistics and correlations for all variables.

	*M*	*SD*	1	2	3	4
1. PE	17.62	14.281	1			
2. MPAT	37.441	7.797	−0.158**	1		
3. SC	67.282	9.329	0.212**	−0.607**	1	
4. PEA	63.308	8.954	0.338**	−0.138**	0.248**	1

N = 1,433. PE, physical exercise; MPAT, mobile phone addiction tendency; SC, self-control; PEA, physical exercise atmosphere, **p < 0.01.

### Moderated Mediation Model Test

**FIGURE 2:**
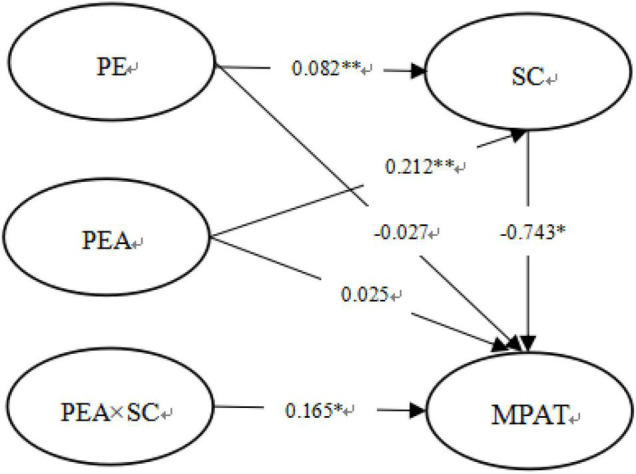
Moderated mediation model.

In this study, the structural equation model was used to test the relationship between PE, SC, PEA, and MPAT among university students, and the item packaging strategy recommended by Wu Yan and Wen Zhonglin was used to pack all the scales in this study, and then the maximum likelihood method was used to estimate and to test the overall model (Yan and Zhonglin, 2011). According to the steps of moderated mediating test recommended by Wen and Ye (2014), the test was conducted. In this study, the mediating effect and moderating effect were tested simultaneously, and the results were shown in Figure 2. The model fitting indexes are: RMSEA = 0.040, CFI = 0.995, NNFI = 0.977, SRMR = 0.021, and the results show that the data fit the model well (Wen and Ye, 2014). Firstly, PE positively predicted SC (β = 0.082, *t* = 3.748, *p* < 0.01), and SC significantly negatively predicted MPAT (β = –0.743, *t* = –19.929, *p* < 0.01). Bootstrap method was further used to test the mediating effect of SC. The results show that the 95% confidence interval does not include 0. Therefore, SC is a mediating variable between PE and MPAT. Hypothesis 2 was verified. Secondly, after adding SC as a mediating variable, PE did not significantly negatively predict MPAT (β = –0.027, *t* = –1.257, *p* > 0.05), but before adding SC as a mediating variable, PE significantly negatively predicted MPAT (β = –0.158, *p* < 0.05). The results showed that SC played a complete mediating role in the relationship between PE and MPAT. Thirdly, the interaction term of PEA and SC could significantly positively predict MPAT (β = 0.165, *t* = 2.545, *p* < 0.05). Therefore, the PEA moderates the relationship between SC and MPAT, that is, the influence of PEA on MPAT of university students is a moderated mediating effect. Hypothesis 3 is verified.

**FIGURE 3:**
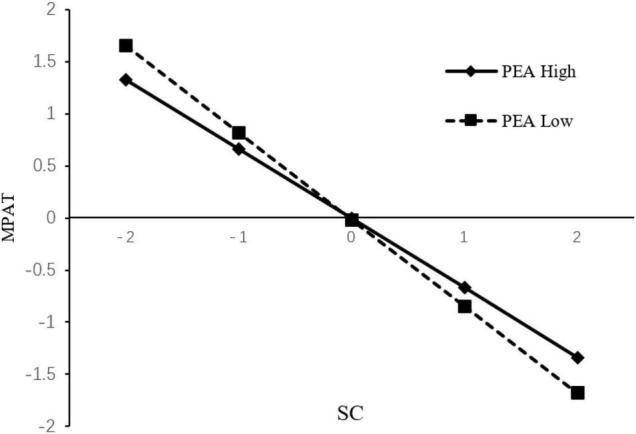
PEA moderates the relationship between SC and MPAT.

Another focus of this study is how the PEA regulates the relationship between SC and MPAT. Therefore, ± 1 of the Z score of physical exercise atmosphere was taken to draw the interaction effect diagram. The moderating effect of physical exercise atmosphere on the relationship between self-control and mobile phone addiction tendency is shown in Figure 3. Simple slope test (Dearing and Hamilton, 2006) showed that in the group with high PEA, SC had a significant negative predictive effect on MPAT (β = –0.67, *t* = –14.41, *p* < 0.01). An increase of 1 standard deviation of SC would reduce MPAT by 0.67 standard deviation. However, in the group with low PEA, the negative predictive effect was stronger (β = –0.83, *t* = –15.02, *p* < 0.01), with the increase of 1 standard deviation of SC, MPAT was decreased by 0. 83 standard deviation. Compared with the group with high PEA, the extent of reduction was increased. In general, with the increase of SC level, university students in the group with high PEA were less likely to become addicted to mobile phones than those in the group with low PEA. However, this trend was reversed at the intersection point in the figure, that is, individuals with high SC extremes were more likely to become addicted to mobile phones in the high PEA than in the low PEA.

## Discussion

### Physical Exercise and Mobile Phone Addiction Tendency

According to the above research results, PE significantly negatively predicts MPAT of university students, which verifies hypothesis 1, and is similar to the previous research results (Yanli, 2014; Yuhang, 2019; Xiaoqiang, 2020), that is, the higher the level of PE, the lower the degree of MPAT is. The results show that PE is a positive factor to predict MPAT, and has the cross-age characteristics. According to the theory of Sports Sociology, individual behavior is influenced by both individual psychology and external environmental factors, with the latter factor more decisive. The theory of Ternary Interaction holds that environment, individual and behavior influence each other, and the magnitude of their influence depends on the nature of environment and behavior. PE is a very important external environmental stimulus, which can not only effectively improve the physical health of individuals, but also have a significant effect on mental health and social adaptation (Liwei and Zhixiong, 2003). Research found that individuals in the process of PE, the excitement of the cerebral cortex will be transferred with the original excited cells are suppressed. Therefore, individuals with MPAT may decrease the activity of cells in the brain that use mobile phones and focus their attention on something else when they participate in PE. In addition, physical exercise can also strengthen the nerve-humoral system regulation ability, improving the speed when individual reacting to the eternal environment as well as the ability of adapting to the environment. Therefore, regular participation in PE helps to internalize the correct values, to enhance the happiness of life, to break away from the virtual network world and to develop a positive and healthy lifestyle. In particular, young people should regularly participate in group sports programs. By experiencing different roles, they would be able to deal with the relationship among individuals and groups, to cultivate the virtues of teamwork, to develop the ability to communicate with others. Studies have shown that regular PE can relieve depression, anxiety and other adverse mood states (Hong and Hongli, 2012). Therefore, we should pay more attention to university students’ PE to improve their interpersonal skills, and to provide a good sports environment to meet their basic psychological needs to participate in PE so as to prevent them from being addicted to the internet and falling into MPAT.

### The Mediating Role of Self Control

The research shows that PE can significantly positively predict the SC of university students, which confirms that there is a close relationship between PE and SC. In PE, in order to anticipate the intention of partners and opponents, to cooperate with partners and to adjust task demands, individuals need to use more complex cognitive functions (Tomporowski et al., 2008). Studies have shown that collective physical activities can effectively improve individuals’ SC (Davis et al., 2011). Individual implementation of this cognitive function requires executive control ability to modify cognitive plans to meet changing task requirements (Banich, 2009). Therefore, regular PE can improve individuals’ executive controlling ability (Fengshu, 2015). The study also found that SC had a significant negative predictive effect on university students’ MPAT. The stronger the individual’s SC is, the higher the ability of problem solving and emotional adjustment is, and the lower the degree of MPAT is (Songli et al., 2015). An important finding of this study is that SC plays a complete mediating role between PE and university students’ MPAT, which verifies Hypothesis 2. This result reveals a very important mechanism through which PE affects university students’ MPAT–the role of SC. Studies have shown that the stress coping style and the satisfaction of basic psychological needs are important factors affecting MPAT (Chengfu et al., 2012; Baojuan and Qing, 2016). According to the “Loss of Compensation Hypothesis,” due to the immature mental development and weak emotional regulation ability as well as SC, the adolescent university students are less likely to be able to satisfy their independent needs, interpersonal needs and relationship needs in real life, thus taking “pathological” compensation such as MPAT to meet their needs. However, if their SC is improved, it may reduce the degree of their MPAT (Songli et al., 2015). This suggests that the level of university students’ PE can be promoted to improve their SC and to reduce their MPAT.

### The Moderating Role of Physical Exercise Atmosphere

This study also confirmed that the PEA moderated the relationship between SC and MPAT among university students, and verified Hypothesis 3. In general, under different SC levels, the degree of MPAT in the high PEA is generally lower than that in the low PEA. According to the self-determination theory, this result may be caused by the fact that the positive PEA can significantly predict the satisfaction of individual basic psychological needs (Joe et al., 2017). A good PEA and sufficient interpersonal support can improve an individual’s prosocial behavior and self-esteem (Binbin and Dan, 2009; Yan et al., 2018), while good and convenient sports venues and beautiful natural environment can predict an individual’s exercise motivation (Kelei, 2019). Therefore, it is helpful for individuals to develop good sports habits and to reduce mobile phone addiction tendency. However, poor PEA can hardly satisfy the basic psychological needs of individuals, which will promote the problem behaviors of individuals (Dan et al., 2013). In conclusion, a good PEA can bring about the satisfaction of individual growth needs and effectively reduce MPAT, which is an important protective factor for individual growth. However, the study also found a slightly unexpected result, that is, if the individual is in a state of extreme self-control, high PEA can actually increase the degree of their MPAT (Figure 3). On the one hand, this result can be explained by the stress vulnerability model, that is, when the risk (SC) reaches a certain level, the protective factor (PEA) may lose its ability to resist the risk (Dongping et al., 2012) and cannot play a protective role. On the other hand, according to the theory of Extreme SC, in the process of extreme SC, individuals will easily fall into self-denial and no longer engage in positive self-dialogue and thus deny their own efforts. At the same time, individuals with extreme SC tend to deny themselves and not to adjust the goals to avoid mistakes. If emotions can not be effectively handled, some negative emotions may occur such as emptiness, anxiety, depression and other psychological problems in the long run. In the low PEA, the SC performance of university students is relatively normal, but in the high PEA, they tend to have a stronger sense of emptiness, boredom. When this occurs, they are more likely to use Internet to fight against those negative moods, leading to MPAT. This indicates that when we intervene on these university students, the improvement of PEA should not be the only factor that is stressed, keeping their SC level at an appropriate level is also of great importance to reduce their MPAT.

## Limitations and Prospectives

This study suggests the relationship between PE and MPAT among university students, and constructs a moderated mediation model, which has important theoretical value for understanding the causes of university students’ MPAT, and meanwhile provides enlightenment for the prevention and intervention of university students’ MPAT. First of all, schools should pay full attention to individual PE by creating PEA and organizing after-school physical activities to provide students with more PE opportunities. Parents should care about the physical and mental health of their children, spending more time on accompanying children to participate in PE. Secondly, community, school and family should be integrated organically to improve the level of SC of university students. Finally, PEA should be developed to provide the necessary human resources, natural resources, information resources and conditions resources to satisfy university students the basic sports psychology needs. At the same time, the students of extreme SC should be paid more attention targeted measures should be taken when necessary to ensure that every university students can get better development.

Undoubtedly, this research has yet to be further improved: First of all, this study proved that SC played a complete mediating role between PE and MPAT. As is known to all, whether fully mediation or partial mediation depending on whether the direct effect significantly, and direct effect significantly affected by the sample size, the larger the sample size, the easier it will be significant. Therefore, complete mediation may become partial mediation when the sample size is large enough. In addition, the expression of complete mediation does not mean that there is only one intermediary variable. In fact, there may be many such variables, and researchers should actively explore more similar variables. Secondly, this study is a predictive study, so no causal inference can be drawn. In the future, experimental research design can be used to more effectively explain the influence of PE on MPAT of university students. Thirdly, the samples selected in this study are part of university students, and the sample source is relatively single. It is necessary to be cautious when the results of the relationship between PE, SC, PEA, and MPAT variables are extended to other age groups, and the sample size can be expanded in the future to obtain a broader representation. Finally, the self-report method is used in this study to investigate university students. The common method deviation may interfere with the research results. In the future, data can be obtained from multiple channels such as teachers, peers and parents’ evaluation, so as to further explain the relationship between various variables.

## Conclusion

(1) PE has a significant negative predictive effect on university students’ MPAT; (2) SC plays a complete mediating role in the relationship between PE and college students’ MPAT; (3) PEA moderates the relationship between SC and MPAT among university students.

## Data Availability Statement

The original contributions presented in the study are included in the article/supplementary material, further inquiries can be directed to the corresponding authors.

## Ethics Statement

This study was performed in accordance with the Declaration of Helsinki, and was approved by the Institutional Review Board of the School of Physical Education and Health at Zhaoqing University of China and the School of Physical Education at Huaibei Normal University of China, and all participants signed an informed consent form and were paid for their participation. The patients/participants provided their written informed consent to participate in this study.

## Author Contributions

K-LG and Q-SM: conceptualization, methodology, formal analysis, and writing – original draft preparation. Q-SM: software. Q-SM, S-JY, and ZH: validation. K-LG, Q-SM, S-JY, CL, ZH, and JJ: investigation. Q-SM, S-JY, CL, ZH, JJ, and XL: resources. Q-SM: data curation. K-LG, CL, JJ, and XL: writing – review and editing. K-LG, JJ, and XL: visualization, supervision, and funding acquisition. K-LG: project administration. All authors contributed to the article and approved the submitted version.

## Conflict of Interest

The authors declare that the research was conducted in the absence of any commercial or financial relationships that could be construed as a potential conflict of interest.

## Publisher’s Note

All claims expressed in this article are solely those of the authors and do not necessarily represent those of their affiliated organizations, or those of the publisher, the editors and the reviewers. Any product that may be evaluated in this article, or claim that may be made by its manufacturer, is not guaranteed or endorsed by the publisher.
